# Characteristics of Open-Graded Friction Course Macrotexture and Macrostructure and Its Effect on Skid Resistance under Rainfall

**DOI:** 10.3390/ma17184658

**Published:** 2024-09-23

**Authors:** Liang Song, Di Yun, Wei Ye, Jie Gao

**Affiliations:** 1 School of Traffic and Transportation Engineering, Xinjiang University, Urumqi 830017, China; slpl@sohu.com; 2 Xinjiang Transportation Investment Construction Management Co., Ltd., Urumqi 830099, China; yewei1987highway@163.com; 3 Key Laboratory of China’s Transportation Industry for Highway Engineering Technology in Arid Desert Region, Urumqi 830099, China; 4 School of Automobile and Traffic Engineering, Wuhan University of Science and Technology, Wuhan 430081, China; 5 School of Civil Engineering and Architecture, East China Jiaotong University, Nanchang 330013, China; gaojie@ecjtu.edu.cn

**Keywords:** skid resistance, surface macrotexture, internal macrostructure, contact depth, semantic image segmentation

## Abstract

An Open-Graded Friction Course (OGFC) presents a rough surface and a porous structure and provides skid resistance under wet conditions, differing from that of a dense graded mixture. This study explored the distribution of surface macrotexture with depth in OGFC. Using cross-sectional images and semantic image segmentation techniques, the internal structure, porosity, and void size distribution were analyzed to assess the effectiveness of rainfall drainage. Skid resistance was evaluated with a British Pendulum Tester, focusing on the influence of surface macrotexture and internal macrostructure, particularly with regard to contact depth. Results show that finer gradations increase surface roughness peaks, which are concentrated near the top surface. In contrast, coarser mixtures exhibit a greater effective contact depth and more peaks with higher curvature. Finer gradations also result in lower porosity, greater void dispersion, and smaller average void diameters. During heavy rainfall, OGFC-13 exhibits the highest friction coefficient due to its effective contact, surface roughness, and internal voids, which facilitate water expulsion. This research provides insights into the skid resistance mechanism of OGFC in wet conditions and offers practical guidance for selecting the optimal gradation.

## 1. Introduction

Hydroplaning is highly likely to occur when a vehicle travels at high speeds on wet pavement [1,2], posing a serious risk to both driver and passengers. Research indicates that, under wet conditions, the macrotexture of a dense asphalt mixture surface acts as a drainage channel beneath the tire, allowing direct contact between the tire and the road surface, thus enhancing skid resistance [3]. However, surface water cannot be discharged quickly enough at high speeds, resulting in significant hydrodynamic pressure that prevents the tire from maintaining contact with the pavement, thereby reducing friction [4,5].

When a dense graded mixture is exposed to rainfall, a water film forms on the pavement surface [6]. The water film thickness, typically calculated based on the amount of water sprayed per unit area, represents the surface moisture level [7]. Ji et al. and Ma et al. introduced sensors to measure water thickness on the surface and used Mean Profile Depth (MPD) and Mean Texture Depth (MTD) to assess the effect of macrotexture on drainage [8,9]. However, for an open-graded friction course (OGFC), which has voids ranging from 18% to 25%, surface water can permeate into the pavement through these voids [10], altering the water film formation and its impact on skid resistance. Wang and Xiao et al. demonstrated that the drainage capacity of porous asphalt pavement is influenced not only by the road surface slope, pavement layer thickness, and base material type, but also by the porosity [11,12]. Therefore, for asphalt mixtures with a porous structure, it is crucial to study their internal void drainage efficiency and its impact on skid resistance performance.

Research on pavement surface roughness theoretically demonstrated that tread rubber only contacts the top layer of the surface texture [13,14]. Yun et al. proposed a method to obtain the contact depth based on the measured contact area and bearing area ratio curve. Their findings revealed that considering pavement texture within the contact depth range could improve skid resistance prediction [15]. This suggests that parameters calculated using the full-depth texture of the surface do not represent the texture characteristics within the actual contact area. Guo’s study supported this view and proposed that the directional difference in pavement texture should also be considered [16]. For dense mixtures, the pavement texture serves a dual purpose: facilitating water drainage and providing contact with the tire rubber. An increase in the part of the texture for drainage corresponds to a decrease in the surface area available for contact. Consequently, even in the deeper part with absence of contact, the macrotexture plays a crucial role in expelling water. On open-graded surfaces, however, with a porosity range of 18–25%, water is primarily drained through the mixture’s internal voids rather than being expelled through the surface texture [17]. In this instance, the internal voids of the pavement primarily handle water drainage, while the macrotexture of the road surface mainly ensures contact with the tire rubber. This difference results in distinct skid resistance mechanisms between open-graded and dense-graded pavements under wet conditions.

To better understand the influence of internal structure on drainage performance, researchers have used Computed Tomography (CT) scanning and image processing technology to examine the voids. Zhou et al. investigated the effect of pore characteristics on the clogging risk of pervious concrete and proposed equivalent diameters of pores [18]. Shan et al. studied the number and the size distribution of pores, finding that an increase in porosity, a decrease in tortuosity, and an increase in seepage channels lead to higher permeability [19]. Jiang et al. explored void spatial distribution inside the mixture [20,21], proposing parameters such as pore number and pore connectivity to characterize the macrostructure inside the porous mixture that can facilitate the drainage of surface water. It was found that porosity and connectivity are significantly affected by the passing rate and the maximum nominal aggregate size [22]. Pei and Zhang explained the correlation between macrostructure and water permeation performance using statistics and topological principles [23].

However, the interaction between the structure of the open-graded asphalt mixture and the surface texture in affecting skid resistance under rainfall conditions remains unclear. With advances in computer and sensing technology, using images to predict engineering quality in pavement detection has become one of the most intuitive and effective methods [24]. Dong et al. used red–green–blue images to reconstruct the pavement macrotexture based on deep convolutional neural networks [25]. Chen utilized deep learning semantic image segmentation techniques, including Res-UNet, ED-SegNet, and G-Enet to recognize the meso-structure of permeable concreate. The results showed that these methods outperformed conventional techniques in efficiency, recognition accuracy, and the ability to distinguish and identify aggregate, pores, and cement binders [26].

In summary, unlike the macrotexture in dense graded mixtures, which serves both as a drainage channel and provides contact with tread rubber, the macrostructure of open-graded mixtures, due to its large void structure, primarily provides contact with the rubber. This results in different factors affecting skid resistance performance under wet conditions between the two types of mixtures. Currently, the depth of the part that comes into contact on the rough surface of OGFC with rubber is unknown, and the relationship between the internal macrostructure and its drainage is unclear, resulting in an incomplete understanding of the skid resistance mechanism. Therefore, this paper proposes considering the contact depth on the basis of the pavement texture and using semantic image segmentation technology to identify the internal structure, revealing the influence of both surface texture and internal structure on the OGFC mixture.

## 2. Specimen Preparation

### 2.1. Asphalt and Modifier

A high-viscosity asphalt modifier, TAFPACK-Super (TPS, TAIYU, Hanoi, Vietnam), was used to modify road asphalt from the Republic of Korea (SK-70, SK Holdings, Seoul, Republic of Korea). The primary technical properties of the modified asphalt were tested according to the Standard Test Methods of Bitumen and Bituminous Mixtures for Highway Engineering JTG E20-2011 [27], and the results are presented in Table 1.

### 2.2. Aggregate, Gradation, and Specimen Preparation

**Figure 1:**
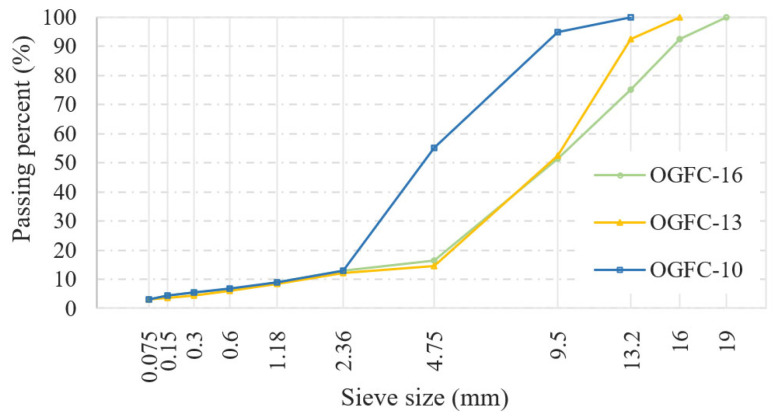
Gradation of the OGFC mixture.

Limestone aggregate from Shaanxi Lianhuasi (Xi’an, China) stone material factory was used to prepare the specimens. The coarse aggregate had a crushing value of 22% and a flat-elongated particle percentage of 8%, both of these meeting the requirements outlined in the Technical Specifications for Construction of Highway Asphalt Pavement JTG F04-2004 [28]. The tests were conducted in accordance with the Test Method of Aggregate for Highway Engineering JTG 3432-2024 [29]. The gradation is shown in Figure 1, and the optimal asphalt content for OGFC-10, OGFC-13, and OGFC-16 was determined to be 5.2%, 4.9%, and 4.7%, respectively.

Specimens were prepared using the wheel rolling compaction method in compliance with the Standard Test Methods of Bitumen and Bituminous Mixtures for Highway Engineering JTG E20-2011. The vacuum density tester measured void ratios as 16.5%, 19.4%, and 21.8%, according to the JTG E20-2011 [27]. To investigate the internal macrostructure, specimens with the same gradation were prepared using the gyratory compaction method, ensuring the same porosity as achieved with the wheel rolling method by controlling the compaction height.

## 3. Macrotexture and Macrostructure of Specimens

### 3.1. Measurement and Evaluation of Macrotexture

#### 3.1.1. General Procedure

**Figure 2:**
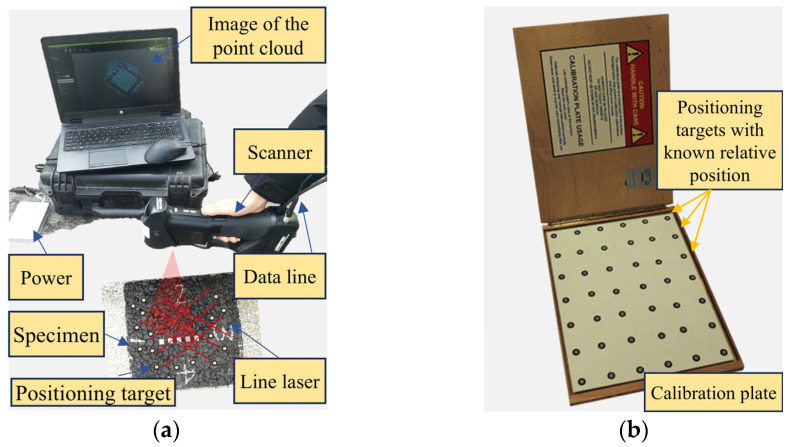
Creaform HandySCAN 300 3D scanner. (**a**) Main parts of the scanner, (**b**) the calibration plate.

A portable 3D scanner (HandySCAN 300, Creaform, Lévis, QC, Canada) was used to scan the specimen and capture the three-dimensional surface texture, as shown in Figure 2. The scanner offers a resolution of 0.1 mm and an accuracy of 0.04 mm. Before scanning, positioning targets were strategically placed on the surface of the specimen to record their relative positions based on the geometrical relationship of the angles of departure and arrival in the scanner (Figure 2a). These positioning targets serve a similar purpose to benchmarks in surveying. When the laser scans the pavement surface and the field of view includes four or more positioning markers, the software calculates the elevation of the corresponding positions based on the predetermined sampling interval. A calibration using a plate with positioning targets of known relative positions (Figure 2b) is required if there is a considerable time interval between scans or significant environmental changes. After obtaining the surface macrotexture data, the form and roughness out of the range of the pavement macrotexture were removed and filtered, with upper and lower wavelength limits set to 50 mm and 0.5 mm, respectively.

**Figure 3:**
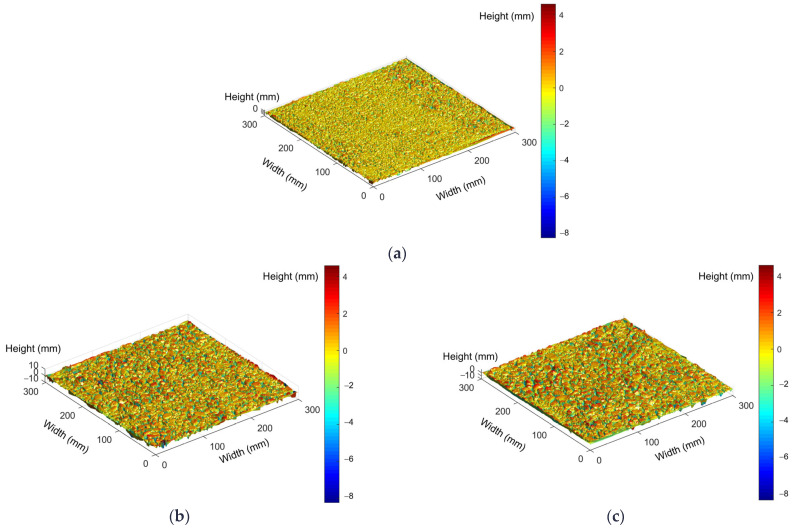
Surface 3D reconstruction images of OGFC specimens. (**a**) OGFC-10, (**b**) OGFC-13, (**c**) OGFC-16.

Figure 3a–c shows the 3D surface data reconstructions of the OGFC-10, OGFC-13, and OGFC-16 specimens, respectively. Noticeable differences in the macrotexture roughness of mixtures with varying gradations are evident. As the maximum particle size decreases, the surface protruding points become denser; additionally, the color bars on the right side of each image in Figure 3a–c are the same; surfaces with colors closer to the upper side of the color bar have the greater height difference. Therefore, larger maximum particle size results in greater height differences between the lowest and the highest points on the OGFC surface.

After filtering and other pre-processing steps on the surface, the mean profile depth (MPD), the average number of peaks in unit area (*Spd*), and the arithmetic mean peak curvature (*Spc*) were chosen to characterize the macrotexture. A higher *Spd* value indicates denser surface texture peaks, while a higher *Spc* value indicates sharper peaks, and vice versa.

**Figure 4:**
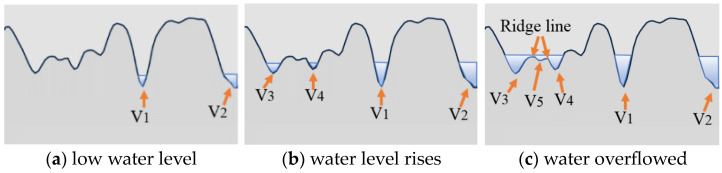
Watershed algorithm to identify valley areas.

The MPD values were calculated as per the specifications of the automated Pavement Condition Survey JTG/T E61-2014 [30]. The watershed method, based on image segmentation technology, was used to identify the peaks on the specimen surface [31]. Specifically, it is assumed that the water rises gradually from the lowest point of the surface, flooding the area below the water plane, as illustrated in Figure 4a. Here, V stands for valley, with the number indicating the specific valley. As water fills an isolated valley and the water level rises, it eventually overflows the current valley. The points where the water surface of different valleys meet form a “ridge line”, which delineates the separate valley areas (Figure 4c). Similarly, the peak areas of the surface can be identified. The watershed algorithm also identifies some small bumps and dips, such as V5 in Figure 4c, as peaks and valleys.

These minor features have little impact on surface performance but can interfere with the calculated characteristic values of peaks and valleys. Thus, wolf pruning was applied to suppress excess segmentation by removing regions with peak height or valley depth below a specific threshold, typically 5% of the maximum surface height.

After identifying the peaks, the density of peaks within the evaluation area can be determined by dividing the number of peaks by the area, yielding the *Spd* value as per Equation (1). For each peak, the two principal curvatures were computed, representing the maximum and minimum normal curvatures. On the surface featuring *N* peaks, the arithmetic mean curvature for a specific peak is computed as the average of the two principal curvatures. Over the entire area, the arithmetic mean peak curvature can be calculated by Equation (2).

(1) $$Spd=\;\frac{N}{A}$$

(2) $$Spc=-\frac{1}{2}\frac{1}{N}\sum_{k=1}^{N}\left(\frac{\partial^{2}z(x,y)}{\partial x^{2}}+\frac{\partial^{2}z(x,y)}{\partial y^{2}}\right)$$

where *N* refers to the number of peaks in a given evaluation area, *A* is the given evaluation area, *k* refers to the number of the *k*th peak, and *z*(*x*, *y*) denotes the surface height at the coordinate (*x*, *y*).

#### 3.1.2. Rubber–Pavement Contact Depth

Given the high roughness of the OGFC surface, the rubber slider of the pendulum tester primarily contacts the shallow top surface [32]. In this study, Persson’s multi-scale contact theory was used to calculate the real contact depth before determining the texture parameters to describe the surface features [33]. These features’ effects on skid resistance were then analyzed. The equation used for the calculation is shown in (3)

(3) $$w=\;h_{\max}-\bar{u}$$

where *w* represents the real contact depth, and *h*_max_ is the highest point on the studied surface area. The $\bar{u}$ is the distance between the bottom of the rubber slider and the lowest point on the surface. $\bar{u}$ and $u_{0}$ are governed by Equation (4). The parameter E* can be calculated by (5), while β and $u_{0}$ depend on the surface roughness power spectrum, and $u_{0}$ can be calculated by the Equations (8)–(10).

(4) $$p=\;\beta E^{*}e^{-\bar{u}/u_{0}}$$

Here, *p* is the pressure at the time of contact. When the pendulum instrument is in contact with the test surface, the forward static pressure is 22.2 N. Combined with the contact area, the pressure *p* between the two is approximately 0.074 MPa.

(5) $$E^{*}=\;E/\left(1-\nu^{2}\right)$$

where *E* is the elastic modulus, approximately 6.0 MPa, and the Poisson ratio ν is 0.5.

(6) $$u_{0}=\sqrt{\pi \gamma }\int_{q_{0}}^{q_{1}}dq\;q^{2}\;C(q)\;W(q)$$

(7) $$W(q)=\;{\left(\pi \int_{q_{0}}^{q}dq^{\prime }\;{q^{\prime }}^{3}\;C(q^{\prime })\right)}^{-1/2}$$

(8) $$C(q)=\;\frac{1}{{\left(2\pi \right)}^{2}}\int d^{2}x\;\langle z(x)h(0)\rangle e^{-iq\cdot x}$$

where, *q* is the wave vector, representing the radian per unit length, *q*_0_ is the cut-off wave vector, roughly equivalent to the nominal maximum particle size of the mixture, and *q*_1_ is the maximum cut-off wavelength. Since *u*_0_ is mainly determined by the roughness of long-wavelength, this study takes *q*_1_ as the maximum resolution obtained by the 3D point cloud of the specimen, which is 0.2 mm. For a surface with a fractal dimension close to 2, where γ is approximately 0.4,  z(x)= z(x,y) is the height of the surface profile relative to the average plane, and $\;\langle \ldots \rangle$ stands for ensemble averaging.

### 3.2. Measurement and Evaluation of Macrostructure

**Figure 5:**
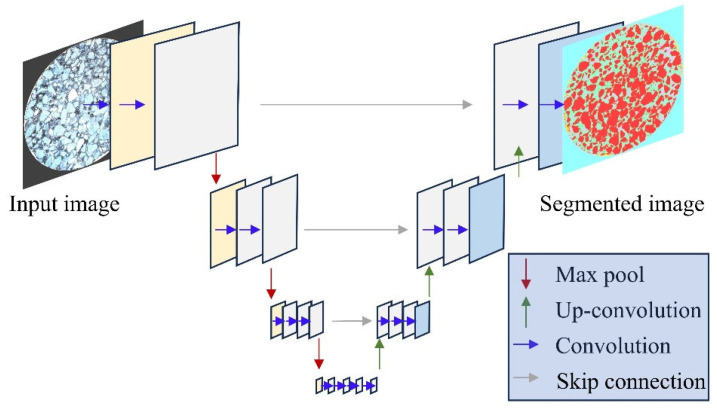
The architecture of U-net for image segmentation.

Specimens prepared using the gyratory compaction method were cut to expose the internal structure. An 8-megapixel camera captured cross-sectional images, such as the input image shown in Figure 5. Each specimen had no fewer than three cross sections, distributed at various specimen levels (upper, middle, and lower).

Using U-Net-based deep learning for semantic image segmentation, different components were segmented into several classes, and relevant void features were extracted. U-Net is a convolutional neural network (CNN) designed for semantic segmentation. The initial convolutional layer sequence in U-Net, combined with the maximum pooling layer, performs down-sampling, progressively reducing the resolution of the input image. These layers are followed by a series of transposed convolutional layers that use up-sampling operators to continuously increase the image resolution.

Deep learning-based semantic segmentation can accurately classify pixels into three categories: aggregate, mortar, and void. It can also identify the black area outside the specimen and the bright area around the edge of the specimen, as shown in the segmented image (Figure 5).

**Figure 6:**
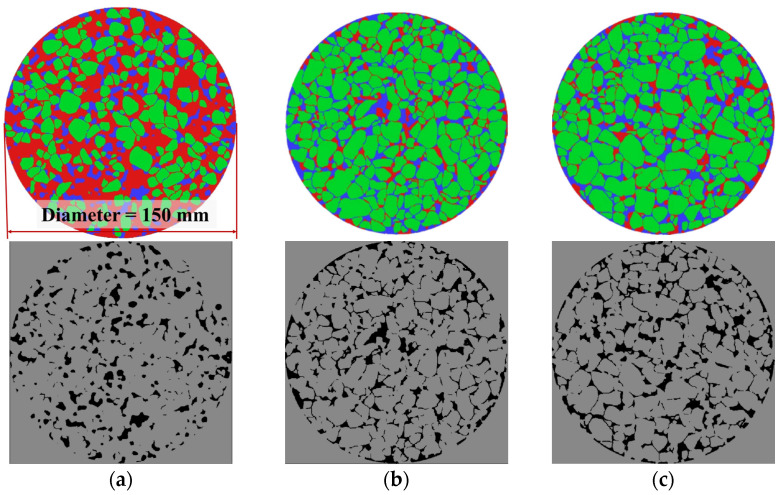
Segmented parts of specimens’ section and corresponding extracted voids, (**a**) OGFC-10, (**b**) OGFC-13, (**c**) OGFC-16. In the top row of the figures, red represents mortar, blue indicates voids, and green denotes aggregates.

After image segmentation, the voids, indicated by the blue area in a cross section of the asphalt mixture, can be extracted as shown in the dark area (Figure 6). The void area of OGFC-10 was the smallest, gradually increasing with the coarser gradation, aligning with the measured porosity. Quantitatively, the void areas were calculated using Equation (9) to assess the internal macrostructure.

(9) $$A_{\mathrm{void}}=\;\frac{N_{\mathrm{pixel-void}}}{N_{\mathrm{pixel-section}}}\cdot 150\cdot 150\cdot \frac{\pi }{4}$$

where, *A*_void_ is the void area, *N*_pixel-void_ is the number of pixels covering the void, and *N*_pixel-section_ is the total number of pixels covering the specimen section.

### 3.3. Skid Resistance Measurement under Different Rainfall Intensities

#### 3.3.1. Control of Rainfall Intensity

A rainfall simulator was employed to ensure uniform rainfall over the specimen. The simulator primarily consists of a graduated water storage bucket, a flow control valve, and a sprinkler. The spray area should completely cover the specimen, and the proportion of water sprinkled on the test specimen can be calculated. Rainfall intensity, defined as the volume of water sprayed on the surface over a given time period, was adjusted by regulating the valve. It is commonly measured in units of depth per unit time. The intensity was calculated using Equation (10).

(10) $$i_{\mathrm{rainfall}}=\;V/S$$

where *i*_rainfall_ refers to the rainfall intensity(mm/h), *V* is the volume of rainfall per unit time (mL/h), and *S* is the area covered by the sprinkling water (mm^2^).

#### 3.3.2. Measurement of Skid Resistance

The number measured by the British Pendulum Tester, the British Pendulum Number (BPN), is used to assess skid resistance performance. The British Pendulum Tester measures the kinetic energy lost when the rubber slider at the bottom of the pendulum head contacts the pavement surface. Tests were conducted once the simulated rainfall intensity reached the specified level, following the specifications of the Field Test Methods of Subgrade and Pavement for Highway Engineering JTG 3450-2019 [34].

After each test, the surface water on the specimen was wiped off with a dry towel, and the specimen was dried at room temperature. These steps were repeated to measure the skid resistance under various rainfall conditions.

## 4. Result and Analysis

### 4.1. Macrotexture on Specimen Surface

#### 4.1.1. MPD of the Different OGFC Specimens

**Figure 7:**
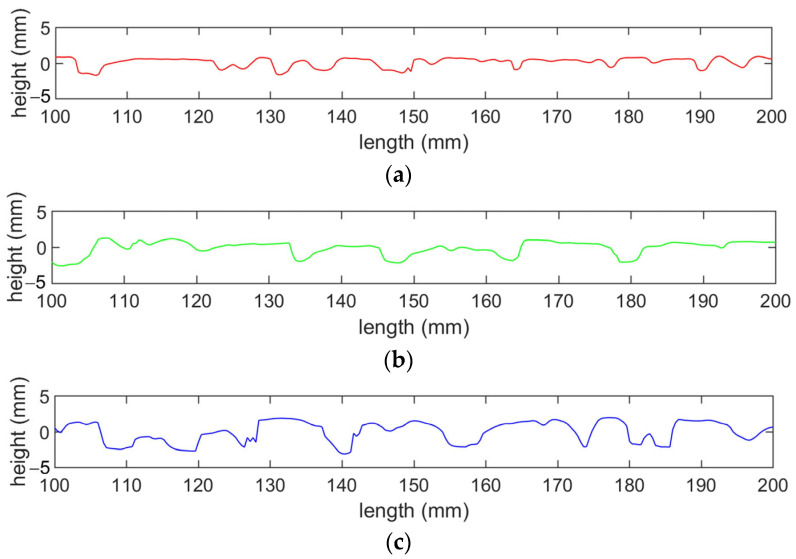
Profiles extracted from different OGFC specimens, (**a**) OGFC-10, (**b**) OGFC-13, (**c**) OGFC-16.

Several profiles, which served as the basis for MPD calculation, were extracted from the three-dimensional surface data points, as shown in Figure 7. The profiles indicate that the height difference between the lowest and highest points on the specimen surface is greater for specimens with larger nominal maximum particle size. The MPD values of the surfaces were calculated using the method described in Section 3.1. The MPD values for the OGFC-10, OGFC-13, and OGFC-16 specimens were 1.96 mm, 2.31 mm, and 2.42 mm, respectively. This trend aligns with the observed height difference on the surfaces of OGFC specimens with varying gradation.

#### 4.1.2. Feature Parameters of the Different OGFC Specimens

Due to the high roughness of OGFC mixtures, rubber sliders do not fully contact the surface [32]. Therefore, this study calculated the number of peaks in unit area, *Spd*, and arithmetical peak mean curvature, *Spc*, at various depths.

(1) Number of peaks per unit area, *Spd*

**Figure 8:**
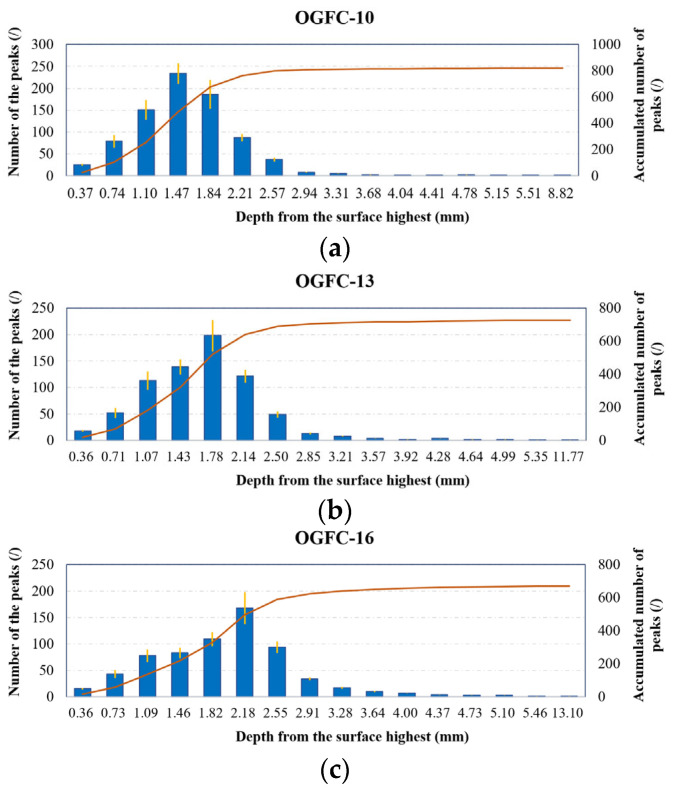
Peak distribution on OGFC surface over the full depth, (**a**) OGFC-10, (**b**) OGFC-13, (**c**) OGFC-16. The orange line represents the variation in the cumulative number of peaks across the calculated intervals.

Figure 8 shows the distribution of the number of peaks within the calculation interval, defined as the space between two horizontal planes. The cumulative number of peaks is the total number above the lower plane of the calculated interval, adjusted to an evaluation area of 0.1 × 0.1 m^2^. The interval length in the subfigures is nearly identical, but differences in maximum height make it challenging to ensure the interval number is an integer with precisely equal length.

As shown in Figure 8, the *Spd* varies significantly on the OGFC surface as the calculation interval changes. The number of peaks per unit area displays a skewed distribution from the highest to the lowest points on the surface, clustering near the top of the surface. The cumulative number of peaks gradually increases with depth from the highest point on the surface, but the rate of increase diminishes until the calculation interval is sufficiently deep.

When comparing OGFC-10, OGFC-13, and OGFC-16 across the entire depth range, OGFC-10 has the highest number of peaks, followed by OGFC-13, with OGFC-16 having the fewest. This indicates that OGFC-10 has the most protrusions on the surface due to its finer coarse aggregate. Consequently, the finer the OGFC mixture gradation, the greater the total number of peaks. This is also the reason why OGFC-13 has an intermediate number of peaks, and OGFC-16 has the fewest.

Moreover, for OGFC-10, OGFC-13, and OGFC-16, at 62%, 45%, and 42% of the whole depth range, respectively, the number of peaks no longer increases. At a depth of 1.4–1.5 mm from the surface, the cumulative peaks of OGFC-10, OGFC-13, and OGFC-16 account for 60%, 45%, and 32% of the cumulative number of peaks on the corresponding surface. This indicates that the peaks in OGFC-10, with the finer gradation and lower profile depth, are more likely to be distributed closer to the surface. In contrast, peaks in OGFC-16 are mainly distributed at greater depth, likely because the higher roughness of coarser gradation causes fine aggregates to deposit deeper within the surface.

(2) Arithmetic mean peak curvature, *Spc*

**Figure 9:**
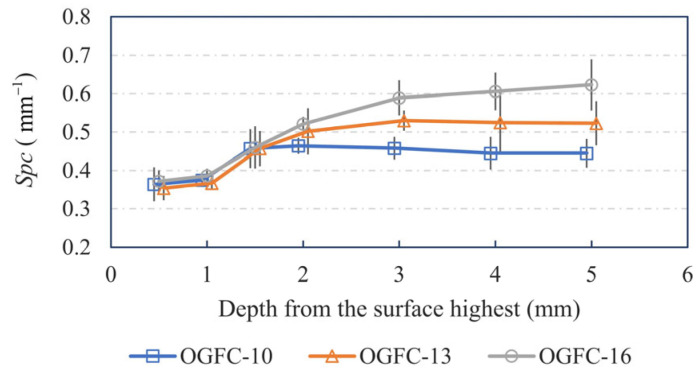
Distribution of the *Spc* of OGFC specimens over the depth.

Figure 9 presents the arithmetic mean peak curvature (*Spc*) values on different surfaces at various calculated depths. Generally, the *Spc* tends to increase with depth. This trend may be attributed to fine aggregates filling the voids between coarse aggregates at deeper surfaces. Fine aggregates, being smaller and sharper, contribute to the increase in *Spc* as the calculated depth range expands.

Additionally, at the same calculation depth, the *Spc* values for OGFC specimens with different gradations increase as the gradation becomes coarser. This finding contradicts the three-dimensional surface data, which suggest that finer gradations result in sharper surface protrusions. The discrepancy might arise because the convex bumps of coarse aggregates are also counted as peaks. These bumps have a smaller diameter than the whole aggregate, leading to calculated *Spc* values that differ from intuitive understanding.

### 4.2. Macrostructure in the Specimen

**Figure 10:**
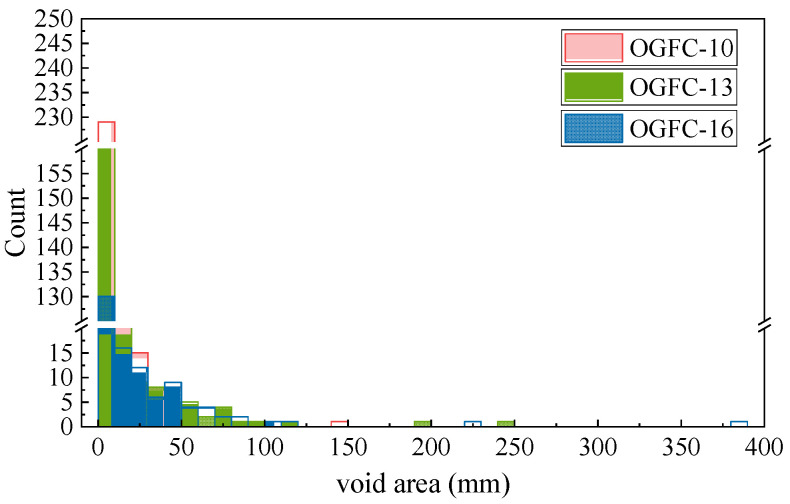
Distribution of void areas in the mixture.

Figure 10 illustrates the internal void area distribution of OGFC mixtures with different gradations. The horizontal axis represents different void area intervals, while the vertical axis indicates the number of voids within each corresponding interval. The number of voids decreases sharply as the void area increases. Additionally, OGFC-10 has the most small voids in the range of 0–10 mm^2^. As the gradation becomes coarser, the number of small voids decreases, while the number of larger voids in the range of 30–120 mm^2^ increases. At this stage, OGFC-16 has slightly more large-area voids than OGFC-13. Combined with the internal macrostructure of the mixture shown in Figure 6, the voids in OGFC-10 are numerous but independent, while OGFC-13 and OGFC-16 have fewer interconnected voids, resulting in a larger single void area.

### 4.3. Skid Resistance under Different Rainfall Intensities

**Figure 11:**
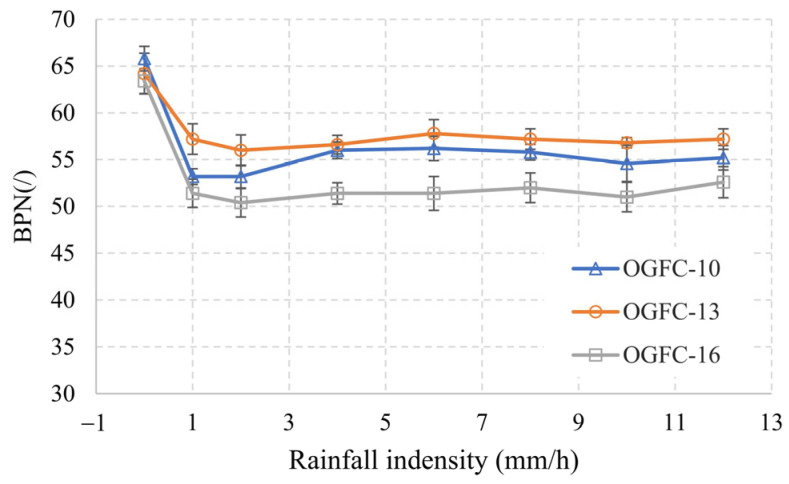
Changes in BPN values of OGFC specimens with rainfall intensity.

Figure 11 shows the changes in BPN values of OGFC specimens with different gradations as the rainfall intensity increases. The horizontal axis represents rainfall intensity, ranging from 0.5 to 11.5 mm/h, from light rain to an extraordinary rainstorm. Generally, as rainfall intensity increases, BPN decreases sharply, initially, reaching a minimum value, then increases slightly, and eventually stabilizes at a value lower than that in dry conditions. This pattern resembles the Stribeck curve, describing friction variation under different lubricant thicknesses, indicating that even though the OGFC mixture is highly permeable, higher rainfall intensity might lead to the formation of a surface water film.

Comparing OGFC-10 with OGFC-13 and OGFC-16 under dry conditions: OGFC-10 has the highest BPN value, though the difference between the three is not significant. As rainfall intensity increases, the BPN value decreases noticeably. The slope of the OGFC-13 curve decreases the slowest, while OGFC-10 and OGFC-16 decrease at similar rates. When the rainfall intensity reaches the rainstorm level, the BPN value of OGFC specimens increase, with OGFC-10 showing the most noticeable increment (about 2 BPN). In contrast, the BPN increments for OGFC-13 and OGFC-16 specimens with coarser gradation are less significant. When stabilized, OGFC-13 has the highest BPN, followed by OGFC-16, with OGFC-10 being the lowest. Contrary to previous research [35], the addition of water increased the BPN values, possibly due to water mobilizing surface contaminants. The measurements here were conducted on a clean surface, suggesting that the accumulated water, a result of inadequate drainage capacity, may have caused this effect.

### 4.4. Relationship between Skid Resistance and Surface Features

Section 4.1.2 reveals that the texture parameters *Spd* and *Spc* vary across different depths, with a significant rate increase at the surface. Therefore, it is essential to consider actual contact conditions and extract the portion involved in real contact between the rubber block and the pavement to study the effect of roughness on skid resistance.

**Figure 12:**
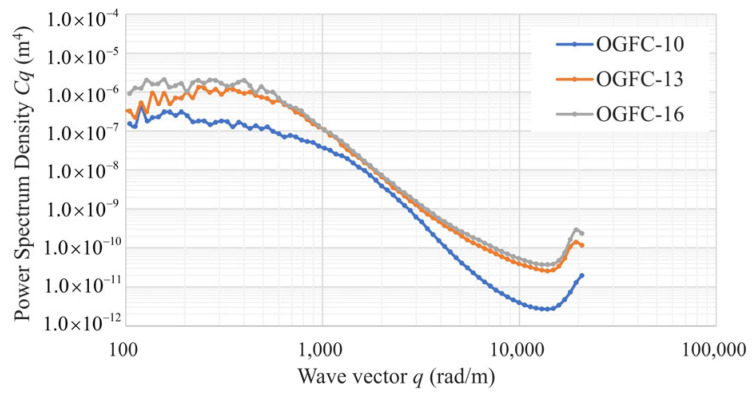
Power spectrum density of the OGFC-10, OGFC-13, OGFC-16 specimens.

**Figure 13:**
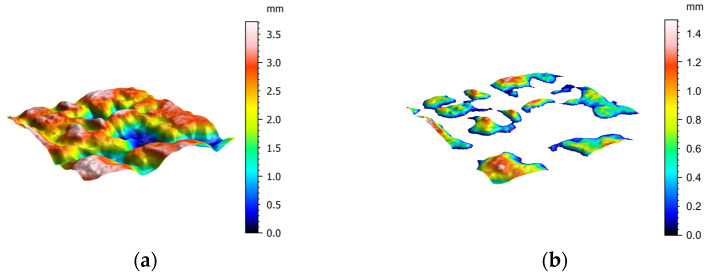
Extracting surface contact area for surface feature parameter calculation. (**a**) Original surface, (**b**) extracted contact areas.

Figure 12 shows the power spectrum density *C*(*q*) of the OGFC-10, OGFC-13, and OGFC-16 specimens. According to Persson’s multi-scale contact theory introduced in Section 3.1.2, the contact depths between the rubber block and the surface of the OGFC-10, OGFC-13, and OGFC-16 specimens are 0.8 mm, 1.3 mm, and 1.5 mm, respectively. The portions of the surfaces that come into contact with the rubber are then extracted from the original surfaces (Figure 13). The pavement texture parameters within these real contact areas are subsequently calculated.

As shown in Table 2, the penetration depth of the rubber block into the specimen surface increases with coarser gradation. Additionally, the *Spd*, indicating the local features of the mixture surface morphology, and the *Spc*, reflecting the sharpness of the peak, both gradually increase. The increment indicates that coarser gradation results in more, sharper roughness bumps in contact.

Compared to OGFC-13 and OGFC-16, OGFC-10 has fewer and flatter peaks. Since friction arises from the adhesion force caused by molecular interactions and the hysteresis force from the deformation of rubber’s viscoelastic materials, the gradual decrease in dry BPN with increasing aggregate size may be due to the effect of adhesive force. The OGFC-10 surface, being relatively flat, forms a larger contact area, providing more adhesive force. Similarly, under dry conditions, the BPN value of OGFC-13 is greater than that of OGFC-16. This suggests that fewer peaks within the real contact depth and flatter peaks result in higher surface friction. This finding contrasts with the established pattern in dense graded mixtures from a previous study [36], where larger aggregate sizes are associated with a significant increase in friction. This discrepancy may be attributed to the rough macrotexture of the surfaces, where contact between the rubber and the specimens enhances skid resistance.

Analyzing the skid resistance data for dry conditions reveals that the friction coefficients are inversely related to the variations in the mean profile depth (MPD) calculated from the actual contact depth. This aligns with the findings of Ahammed and Tighe, who observed that the British Pendulum Number (BPN) on Portland cement concrete (PCC) surfaces follows an upper parabolic trend as texture depth increases [37]. Essentially, beyond a certain MPD value, further increases do not correspond to a proportional rise in BPN value. However, under wet conditions, particularly during heavy rain, the impact of the peaks diminished, and drainage becomes a more significant factor. As rainfall intensity increases, OGFC-13, with a rougher macrotexture compared to OGFC-10, offers greater void space, necessitating more water to fill these areas. At the same time, it provides a larger contact area than OGFC-16 due to its flatter rough bump peaks, resulting in greater friction.

### 4.5. Relationship between Skid Resistance and Macrostructure

As shown in Figure 10, the porosity of OGFC-10 is smaller compared to OGFC-13 and OGFC-16, and the cross-sectional void area is also smaller, indicating smaller porosity and higher dispersion. Assuming the initial speed of rainfall on the road surface is the same, smaller equivalent diameters of these dispersed voids result in slower water discharge, and vice versa. Therefore, the water drainage velocity is not only reduced due to small porosity but also adversely affected by the higher dispersion and smaller average void areas. The significant increase in the BPN value for OGFC-10 when rainfall density reaches the rainstorm level indicates that OGFC-10 has insufficient drainage capacity, leading to the formation of a water film on the surface that hinders rubber movement.

## 5. Conclusions

This study investigated the skid resistance formation mechanism of OGFC mixtures under rainstorm conditions, focusing on both surface macrotexture and internal macrostructure. The following conclusions were drawn:

- The penetration depth of the rubber block into the OGFC specimen must be considered since surface macrotexture features are not uniformly distributed with depth.
- The penetration depth of rubber into the OGFC specimen increases with coarser gradation, leading to a higher number and curvature of peaks in contact.
- Deep learning-based semantic image segmentation is effective for identifying the inner structure of OGFC specimens from the slices of the mixture sections.
- The distribution of voids size within the mixture shows a monotonic descending trend, with finer gradation resulting in more small voids and fewer large voids.
- The surface friction of OGFC asphalt mixtures, as measured by the British Pendulum Tester, changes with rainfall intensity in a pattern similar to the Stribeck curve. However, the BPN value can increase slightly if the mixture provides less drainage capacity than required by the rainfall intensity.

This study applied semantic image segmentation technology based on deep learning to identify internal voids within asphalt mixtures and explored the distribution of feature parameters concerning surface texture depth. This substantiates the need to calculate surface texture parameters by considering contact depth. The analysis of how both surface texture and internal structure influence the skid resistance of OGFC provides a deeper understanding of the mechanisms behind the skid resistance of porous mixtures under wet conditions. Further investigations should consider incorporating the study of microtexture. Although the use of the same aggregate ensures consistent microtexture in the study, variations in microtexture could significantly affect the impacts of macrotexture and warrant future exploration. Additionally, due to the indoor nature of the study and the roughness of the OGFC specimens, conducting the study at high speeds was challenging. While there is a linear correlation between the BPN and the side friction coefficient (SFC60) measured by lateral force coefficient test vehicle, this is not a substitute for high-speed testing. Future work should involve accumulating field data and potentially developing specialized high-speed friction testing equipment for indoor surfaces with pronounced macrotexture characteristics.

## Figures and Tables

**Table 1:** Main technical indicators of modified asphalt.

Modifier Dosage/%	Penetration (25 °C)	Softening Point/°C	Ductility/cm		60 °C Viscosity/(Pas)	Density/ (g/cm^3^)
			5 °C	15 °C		
12	45	87.0	57	>100	137,812	1.03

**Table 2:** OGFC surface macrotexture characteristics and skid resistance.

Mixture Type	Contact Depth (mm)	Parameters Calculated Based on the Extracted Surface			BPN Value		
		*Spd* (/)	*Spc* (/)	MPD (mm)	Max.	Steady	Reduction (%)
OGFC-10	0.8	106	0.37	0.68	65.8	55.5	15.73
OGFC-13	1.3	202	0.43	1.12	64.2	57.3	10.82
OGFC-16	1.5	220	0.46	1.39	63.4	51.8	18.37

Note: *Spd* refers to the number of peaks in an area of 0.1 × 0.1 m^2^.

## Data Availability

The original contributions presented in the study are included in the article, further inquiries can be directed to the corresponding author.
